# High-intensity focused ultrasound ablation assisted using color Doppler imaging for the treatment of hepatocellular carcinomas

**DOI:** 10.1007/s00261-013-0010-z

**Published:** 2013-06-02

**Authors:** Hiroyuki Fukuda, Kazushi Numata, Akito Nozaki, Masaaki Kondo, Manabu Morimoto, Shin Maeda, Katsuaki Tanaka, Masao Ohto, Ryu Ito, Yoshiharu Ishibashi, Noriyoshi Oshima, Ayao Ito, Hui Zhu, Zhi-Biao Wang

**Affiliations:** 1 Gastroenterological Center, Yokohama City University Medical Center, 4-57 Urafune-cho, Minami-ku, Yokohama, Kanagawa 232-0024 Japan; 2 Department of Gastroenterology, Yokohama City University, 3-9 Fukuura, Kanazawa-ku, Yokohama, Kanagawa 236-0004 Japan; 3 International HIFU Center, Sanmu Medical Center Hospital, Naruto167, Sanbu-shi, Chiba 289-1326 Japan; 4 Clinical Development Group, AVS, 1-22-2 Nishishinjuku, Tokyo, 160-0023 Japan; 5 Clinical Center of Tumor Therapy of 2nd Affiliated Hospital, Chongqing University of Medical Sciences, Chongqing, China; 6 Institute of Ultrasonic Engineering in Medicine, Chongqing University of Medical Sciences, Chongqing, China

**Keywords:** High-intensity focused ultrasound, Hepatocellular carcinoma, Ultrasound, Color Doppler imaging

## Abstract

**Purpose:**

We evaluated the usefulness of color Doppler flow imaging to compensate for the inadequate resolution of the ultrasound (US) monitoring during high-intensity focused ultrasound (HIFU) for the treatment of hepatocellular carcinoma (HCC).

**Materials and methods:**

US-guided HIFU ablation assisted using color Doppler flow imaging was performed in 11 patients with small HCC (<3 lesions, <3 cm in diameter). The HIFU system (Chongqing Haifu Tech) was used under US guidance. Color Doppler sonographic studies were performed using an HIFU 6150S US imaging unit system and a 2.7-MHz electronic convex probe.

**Results:**

The color Doppler images were used because of the influence of multi-reflections and the emergence of hyperecho. In 1 of the 11 patients, multi-reflections were responsible for the poor visualization of the tumor. In 10 cases, the tumor was poorly visualized because of the emergence of a hyperecho. In these cases, the ability to identify the original tumor location on the monitor by referencing the color Doppler images of the portal vein and the hepatic vein was very useful. HIFU treatments were successfully performed in all 11 patients with the assistance of color Doppler imaging.

**Conclusion:**

Color Doppler imaging is useful for the treatment of HCC using HIFU, compensating for the occasionally poor visualization provided by B-mode conventional US imaging.

High-intensity focused ultrasound (HIFU), a noninvasive method that can cause complete coagulation necrosis without requiring the insertion of any instruments [1–3], has been applied for the treatment of several human neoplasms [4, 5], including small hepatocellular carcinomas (HCCs) [3, 6–8].

We previously reported that ultrasound (US)-computed tomography (CT) three-dimensional (3D) dual imaging is useful for the treatment of HCCs using HIFU by compensating for the occasionally poor visualization provided by B-mode US [6]. The reason for the poor visualization during HIFU treatment is thought to be that HIFU machines have been designed with the assumption that the US probe will not touch the skin to protect the US probe from the HIFU power during HIFU sonication and the presence of degassed water between the skin and the US probe, consequently, multi-reflections can influence the image quality [6]. Another reason for the poor visualization of HCCs during HIFU treatment is that hyperechos caused by the sonications [9, 10] can obscure the original tumor contour.

The color Doppler method is a blood flow display method using the Doppler phenomenon in ultrasonography [11]. This technique using ultrasonic waves is useful because the direction and velocity, flow rate, and waveform of the blood flow signal can be reliably examined in vivo noninvasively under physiological conditions. This technique was introduced clinically to evaluate the blood flow pattern within tumors during the latter half of the 1980s [12]. Because both the tumor image and the blood flow image can be displayed on a single sonogram using color Doppler flow imaging, information that can otherwise be gained using sonography, hepatic arteriography, hepatic venography, and portography can be obtained simultaneously. Color Doppler sonography can show the blood flow and local hemodynamics in the hepatic vascular system and can be used to evaluate portosystemic shunts [13] and portal vein thrombosis [14]. Color Doppler flow imaging is a diagnostic imaging support system that can provide the location of the tumor in relation to the vessels on the monitor. The purpose of the present study was to evaluate the usefulness of color Doppler flow imaging in compensating for the poor visualization of B-mode US on the HIFU monitor during HIFU treatment for HCC.

## Materials and methods

### Patients

Between July 2007 and October 2008, 26 patients with HCC were treated using HIFU; the treatments in 11 of these patients (7 men, 4 women; mean age, 73.2 years; age range, 65–80 years) were assisted using color Doppler imaging (Table 1). The maximum diameter of the tumors measured on sonography ranged from 10 to 26 mm (mean, 16.5 mm; SD, 4.5 mm). All the patients had Child–Pugh classification A or B liver cirrhosis, a prothrombin time ratio greater than 50%, and a platelet count greater than 50,000/mm^3^. In one patient, the HCC diagnosis was confirmed using a percutaneous needle biopsy. The remaining 10 patients were diagnosed as having HCC based on imaging findings (newly presenting tumor on follow-up ultrasonography in patients with chronic liver disease and characteristic enhancement pattern on contrast-enhanced, multiphase helical CT, contrast-enhanced MRI, or contrast-enhanced US). All 11 patients had liver cirrhosis as a result of hepatitis C. At the time of HIFU, the patients were classified as having Child–Pugh classification A (*n* = 10) or B (*n* = 1) cirrhosis. Transcatheter arterial chemoembolization (TACE), percutaneous ethanol injection (PEI), and rib resection were not performed prior to HIFU treatment. Prior to treatment, the patient’s skin was shaved and degassed using a suction pump. An epidural anesthesia was performed during the procedure. Our hospital ethics committee approved this study, and each patient signed an informed consent form at the time of enrollment.

**Table 1:** Baseline clinical characteristics of the patients with HCC (*n* = 11)

Characteristics	
Number of patients	11
Sex (male/female)	7/4
Age	73.2 ± 4.5 (65–80)
Alcohol/HCV/HBsAg	0/11/0
Child A/B/C	10/1/0
Diameter (mm)	16.5 ± 4.5 (10–26)

### Ultrasound therapy system

Sonications were performed using a clinical US-guided ultrasound surgery system. The Tumor Therapy System (Model AC 0501; Chongqing Haifu Tech Co., Ltd, Chongqing, China) used in this study was guided using real-time ultrasonographic imaging [3]. An HIFU 6150S US imaging unit was installed on the HIFU system to obtain real-time US imaging during HIFU ablation. A 2–5 MHz imaging probe was located at the center of the HIFU transducer and was mounted in a reservoir of degassed water [3]. Therapeutic US energy was produced using a piezoelectric ceramic transducer with a diameter of 20 cm, focus length of 15 cm, and an operating frequency of 1.0 MHz [3]. The focal lesion was ellipsoid, with dimensions of 9.8 mm along the beam axis and 1.3 mm in the transverse direction. The targeted tissue was exposed to acoustic focal peak intensities of 5000 and 15,000 W cm^−2^. A calibrated polyvinylidene difluoride membrane hydrophone with spot diameter 0.5 mm (Shanghai Jiao Tong University, Shanghai, China) was used to map the acoustic pressure field of the focused transducer at peak intensities from 200 to 300 W cm^−2^. After the induction of suitable anesthesia, the patient was carefully positioned either prone or on his or her right side so that the skin overlying the lesion to be treated could be easily placed in contact with the degassed water. We checked for pain and complications, and then the US power was increased gradually until a power of 300–450 W was reached. The time for one sonication was 5 s. During the focused US ablation, the real-time US images obtained before and after each exposure were immediately compared to determine whether the hyperechos, indicating the extent of coagulation necrosis, had covered the desired treatment area [15]. Patients were trained to hold their breath at the point at which their entire tumor became visible when the tumor was located between the ribs or just below the diaphragm.

### Color Doppler imaging

Color Doppler sonographic studies were performed using an HIFU 6150S US imaging unit system and a 2.7-MHz electronic convex probe. On-screen, real-time B-mode, color Doppler imaging, and spectral analysis were simultaneously displayed. In this study, we set that the red indicated flow toward the transducer, and the blue indicated flow away from the transducer. A low wall filter function and a pulse-repetition frequency of 1220 Hz were used to detect tiny vascular structures. If necessary, the pulse-repetition frequency was increased once high-velocity arterial flow was detected. The color gain was adjusted during the examination by selecting the highest value at which the color images were still unaffected by the artifacts. The color signal was used as a guide to obtain the Doppler spectrum. The sample volume used for the spectral analysis was 2–4 mm.

### Assessment of treatment efficacy and follow-up

Dynamic CT scans (Aquilion TSX-101A; Toshiba Medical Systems Corp., Tochigi, Japan) with a section thickness of 5 mm or Dynamic MRI scans (Signa HDX 3.0T system; GE Healthcare, Milwaukee, WI) with gradient-echo (GRE) sequences and T1 fat saturation (TR/TE, 4.8/1.9; flip angle, 12°; matrix size, 320 × 192; section thickness, 4 mm; intersection gap, 0–2 mm; one acquisition) with the bolus injection of a 0.025 mmol/kg dose of gadolinium-ethoxybenzyl-diethylenetriamine pentaacetic acid (d-EOB-DTPA) (Primovist; Bayer Schering Pharma) body weight were performed. Assessments using contrast-enhanced (0.2 mL of Sonazoid suspension; Daiichi Sankyo, Tokyo, Japan) ultrasonography (LOGIQ 7; GE Healthcare, Milwaukee) were performed in all the patients when the hyperecho covered the original tumor and at 1 week after the HIFU procedure. A convex volume 4D3C-L probe (GE Healthcare) was used. All the patients received an intravenous bolus injection of 0.2 mL of Sonazoid via a 24-gage cannula into a forearm vein, followed by 2 mL of a 5% glucose solution and the subsequent infusion of a 5% glucose solution at 10 mL/min. A coded harmonic angio (CHA) mode with a high mechanical index (0.5–0.9) at 8–13 frames per second was selected for the contrast-enhanced 3D US procedure. The focus point was set beneath the tumor [3]. Complete ablation based on the contrast-enhanced ultrasonography findings was defined as a perfusion defect with no enhancement and no tumor vessels during both the early vascular phase and the post vascular phase [16].

## Results

Table 2 summarizes the reasons why the color Doppler images were used. The reasons for the use of the color Doppler images included the influence of multi-reflections and the emergence of a hyperecho. In 1 of the 11 patients, multi-reflections were responsible for the poor visualization of the tumor. In 10 cases, the tumor was poorly visualized because of the emergence of a hyperecho. In these cases, knowing the original tumor location on the monitor by referencing the color Doppler images of the portal vein and the hepatic vein was very useful. Finally, HIFU treatment assisted by color Doppler imaging was successfully performed in all the patients.

**Table 2:** Reasons for use of color Doppler images during HIFU treatment

Reason for color Doppler use	
Multi-reflection	1
Emergence of hyperecho	10

**Fig. 1:**
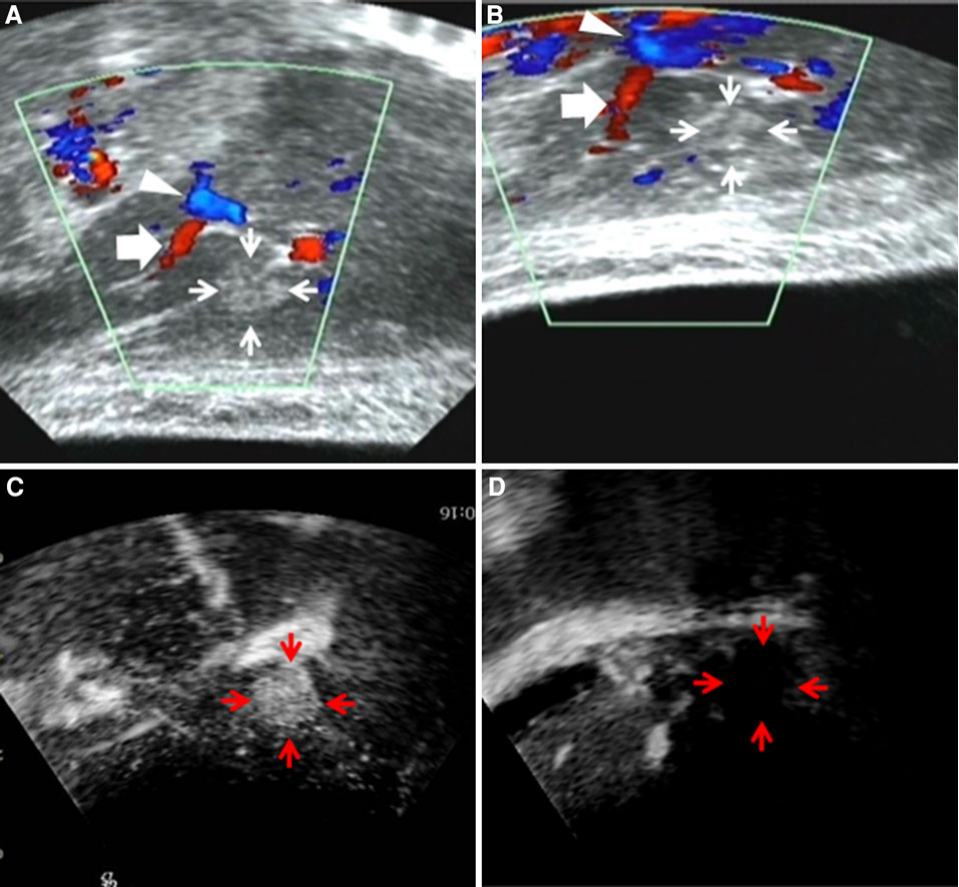
**A** The tumor located in segment 3 (*arrows*) on the monitor ultrasonogram is clearly visible because the US probe was located near the surface of the skin and there were no multi-reflections. The flow of the portal vein is shown *blue signal* (*arrowhead*) and the branch of this portal vein is shown *red signal* (*thick arrow*) before sonication. **B** The US probe was moved downward to focus on the tumor to protect the US probe; as a result, the tumor (*arrows*) on the monitor ultrasonogram was not clearly visible. The flow of the portal vein is shown *blue signal* (*arrow head*) and the branch of this portal vein is shown *red signal *(*thick arrow*) after sonication. By transferring relationship between the color Doppler image data and the original tumor location, the tumor was recognized on the HIFU monitor. **C** Contrast-enhanced US imaging before (*arrows*). **D** Just after HIFU (*arrows*). Just after HIFU, the tumor enhancement disappeared.

**Fig. 2:**
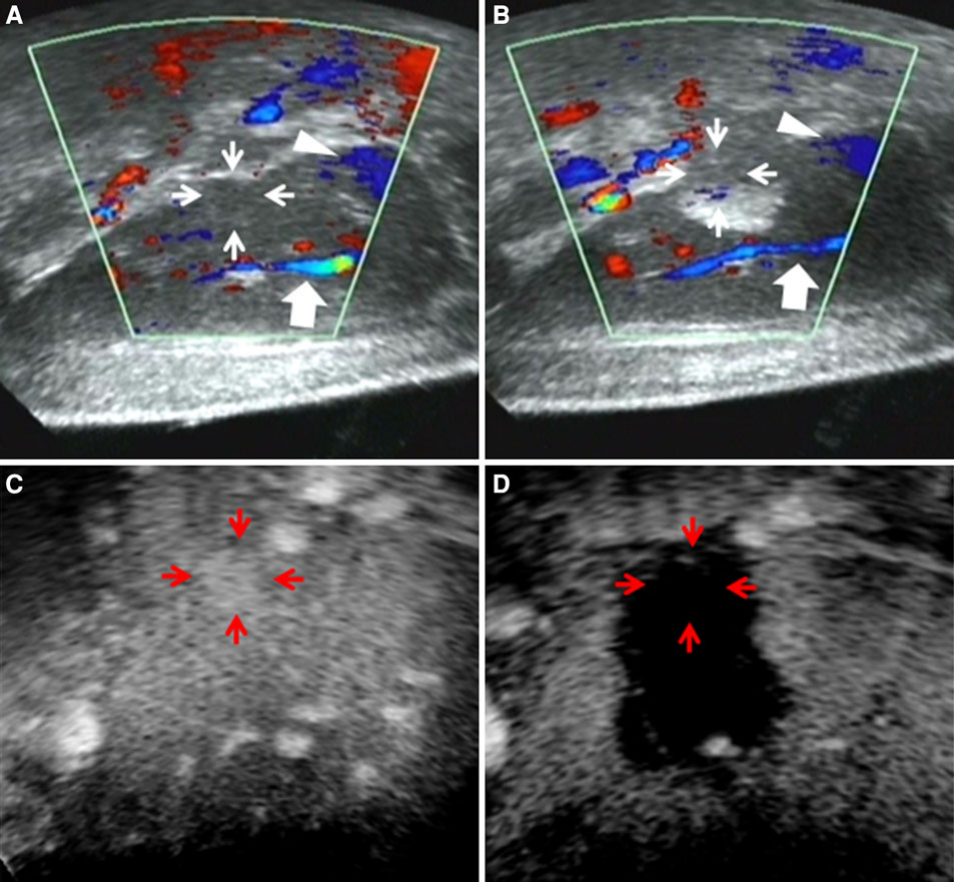
**A** The tumor is located in the posterior surface of the liver (segment 2) (*arrows*) by color Doppler US image before sonication. The flow of the portal vein (*thick arrow*) and the hepatic vein (*arrowhead*) are shown as *blue signal* before sonication. **B** The resulting hyper-echogenic changes obscured part of the contours of the original tumor (*arrows*). The flow of the portal vein (*thick arrow*) and the hepatic vein (*arrowhead*) are shown as *blue signal* after sonication. *Thick arrow* and *arrowhead* indicate the same flow before and after sonication, so, the location of the tumor was recognized after HIFU sonication from the relationship between the color Doppler flow imaging and the original tumor. C The tumor enhancement before the HIFU treatment (*arrows*) disappeared just after the treatment on contrast-enhanced US imaging. **D** The ablated area (*arrows*) is wide enough with an adequate safety margin.

Representative cases of color Doppler imaging in patients with HCC are presented in Figs. 1 and 2. The tumor on the monitor ultrasonogram was clearly visible because the US probe was located near the surface of the skin and no multi-reflections were present (Fig. 1A). The US probe must be pulled down into the inner hole of the transducer to set the focus of the sonication for the tumor to protect the US probe; as a result, multi-reflections between the US probe and the skin surface emerged, and the tumor on the monitor ultrasonogram was not clearly visible. By transferring the color Doppler image data, the location of the original tumor was identified on the HIFU monitor (Fig. 1B). After the HIFU treatment, the tumor enhancement disappeared (Fig. 1C, D), indicating a successful treatment. Figure 2A shows the US monitor with the color Doppler US image of the tumor before the HIFU treatment, showing the tumor to be located on the posterior surface of the liver (segment 2). During the HIFU treatment, this tumor was not clearly depicted on the monitor because of the emergence of a hyperecho after sonication (Fig. 2B). By transferring the color Doppler image data, the location of the tumor was identified on the HIFU monitor (Fig. 2B). The tumor enhancement disappeared after treatment, and the ablated area after HIFU ablation was thought to be sufficiently wide enough to encompass an adequate safety margin (Fig. 2C, D).

## Discussion

In the present study, we used the color Doppler image as an indicator to identify the location of the original tumor because of the presence of artifacts and hyperechos caused by microbubbles that formed during the HIFU treatment.

The B-mode conventional sonography that is used to monitor the HCC during HIFU treatment is sometimes inadequate because of artifacts. We reported that US-CT 3D dual imaging is useful for planning of the HIFU treatment and detecting the tumor, compensating for the occasionally poor visualization provided by the US monitor [6]. The poor visualization using the HIFU monitor is caused by the presence of multi-reflections and/or the emergence of hyperechos during HIFU treatment. The US monitor of the presently used HIFU machine is designed with the assumption that the US probe will not touch the skin, and tumor detection can worsen because the US probe is protected from the HIFU power when it is moved downward, so that tumors located deep within the liver are easily influenced by multi-reflections [6]. Therefore, artifacts cause by the multi-reflections are inevitable; however, the tumor location can be identified in such cases using color Doppler imaging.

Another reason for the poor visualization of HCC during HIFU treatment is that hyperecho caused by the sonications can obscure the original tumor contour [9]. A hyperecho is considered to indicate tissue necrosis and has been used to judge complete ablation [10]. Wang et al. [17] evaluated the relationship between the volume of the ablation and the depth using bovine liver tissue. In the US-guided HIFU therapy system used in the present study, a hyperecho resulting from bubble activity generated during HIFU exposure was regarded as a sign that the treated lesion had been completely coagulated, since the system itself was unable to perform thermometry of the ablated area [15]. The presence of cavitation in the US field was also correlated with a rapid rise in temperature both in vitro [18] and in vivo [19]. The ablated area and the extension of the hyerecho were not always the same, and the original tumor contour was obscure by the hyperecho [6]. The appearance of microbubbles, indicating that the temperature of the area is above the boiling point, was also observed using US during radiofrequency ablation (RFA), making it difficult to identify the location of the original tumor. The evaluation of the ablated area and the hyperecho after RFA was reportedly difficult, and contrast-enhanced ultrasonography was useful for such evaluations [20]. We think that contrast-enhanced ultrasonography may be useful for determining the relationship between the ablated area and the hyperecho at the end of HIFU treatment, but color Doppler may be superior when the location must be confirmed for each sonication during the HIFU treatment, since the patient does not need to be moved. As a result, we were able to clarify the locations of tumors that had been obscured by artifacts based on the relationship between the original tumor and the color Doppler image.

In this study, the utility of color Doppler imaging during HIFU treatment for HCCs was evaluated, and the assistance provided by this modality was found to be useful.
